# Supplementary material for: Plasmon Tuning of Liquid Gallium Nanoparticles through Surface Anodization
Source: Materials (Basel). 2022 Mar 15;15(6):2145. doi: 10.3390/ma15062145 (PMC8948849; doi:10.3390/ma15062145)
Supplement: Supplementary file 1 [file materials-15-02145-s001.zip › materials-1585023-supplementary.pdf]

Supplement

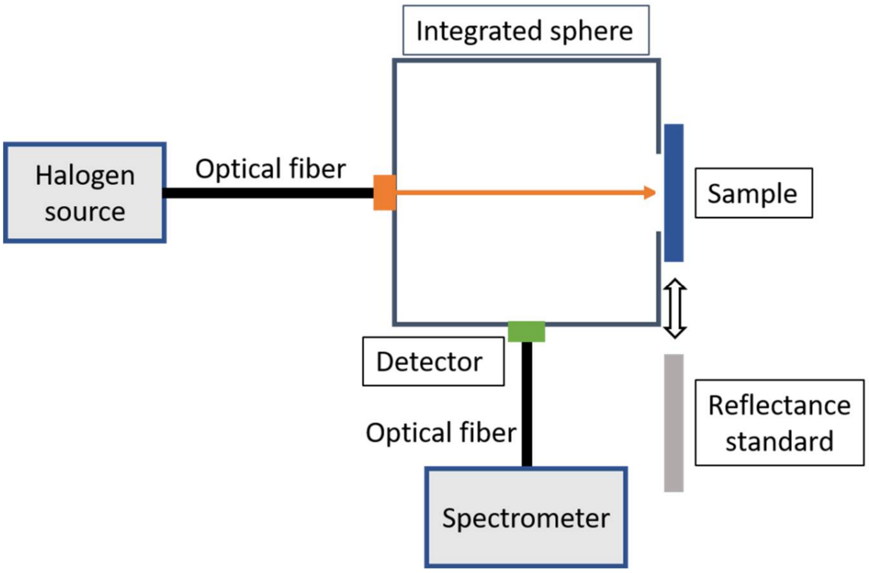

Figure S1. The illustration of the UV–VIS–NIR reflectance measurement setup.

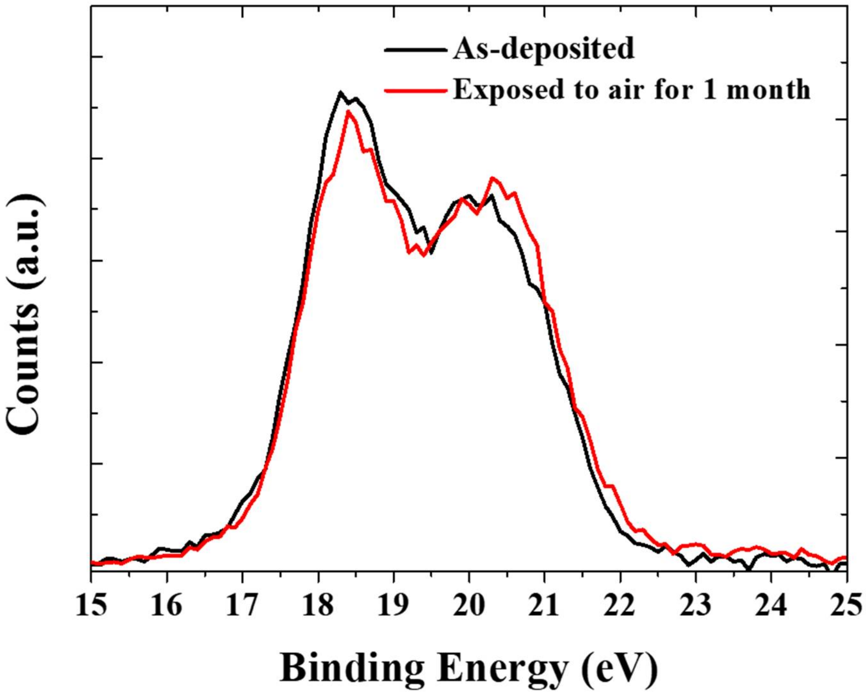

Figure S2. XPS spectra of the 3d level of Ga for as-deposited Ga NPs and the sample after exposed to the ambient atmosphere for a month.

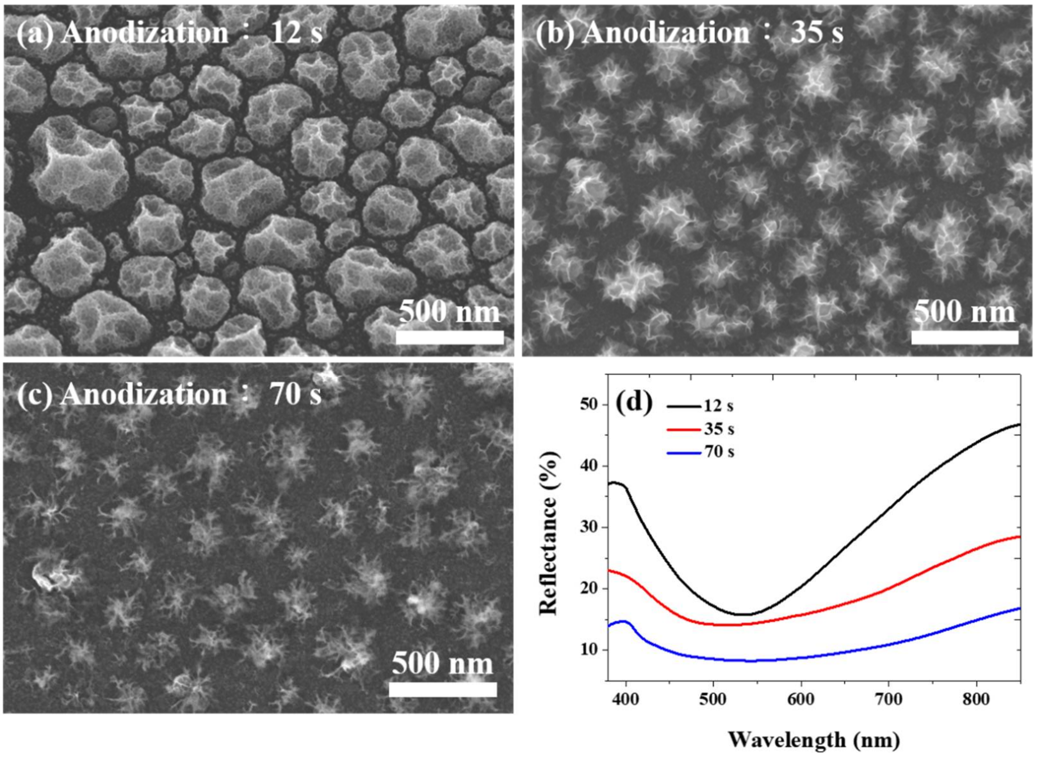

Figure S3. Top view SEM images of Ga-NPs after anodization for (a) 12 s, (b) 35 s and (c) 70 s. (d) Experimental reflectance spectra of representative surface-anodized Ga NPs.

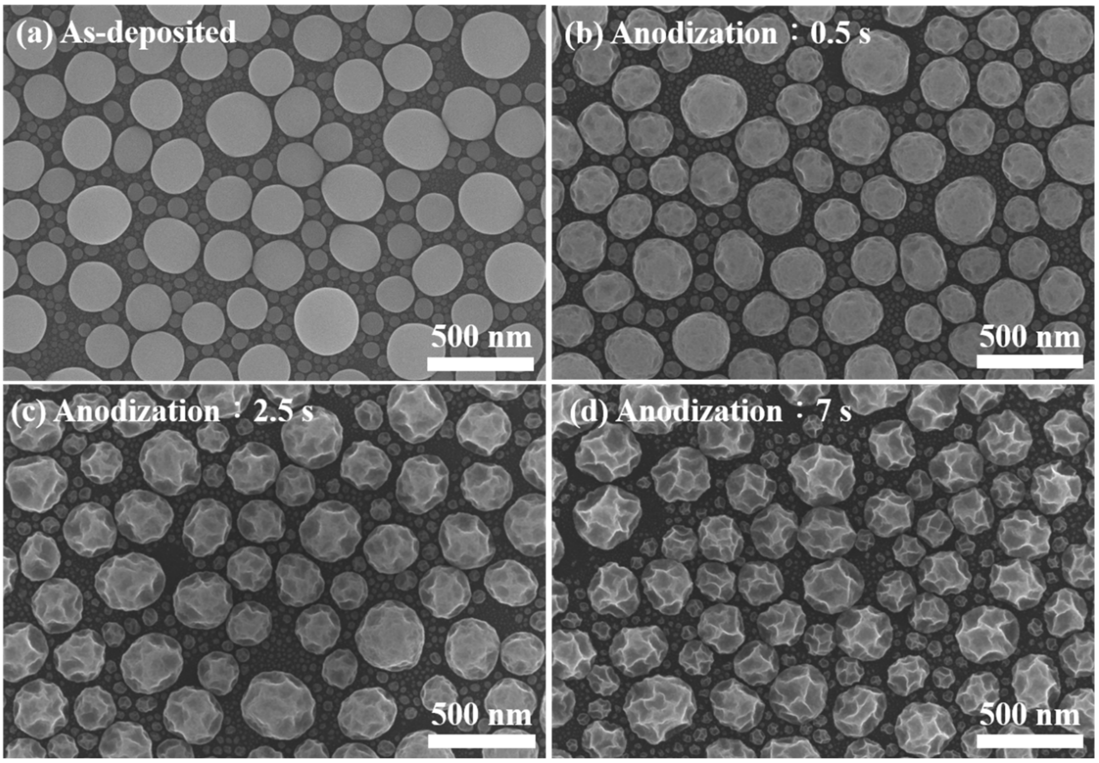

Figure S4. Top view SEM images of Ga-NPs after anodization for (a) 0 s, (b) 0.5 s, (c) 2.5 s and (d) 7 s.

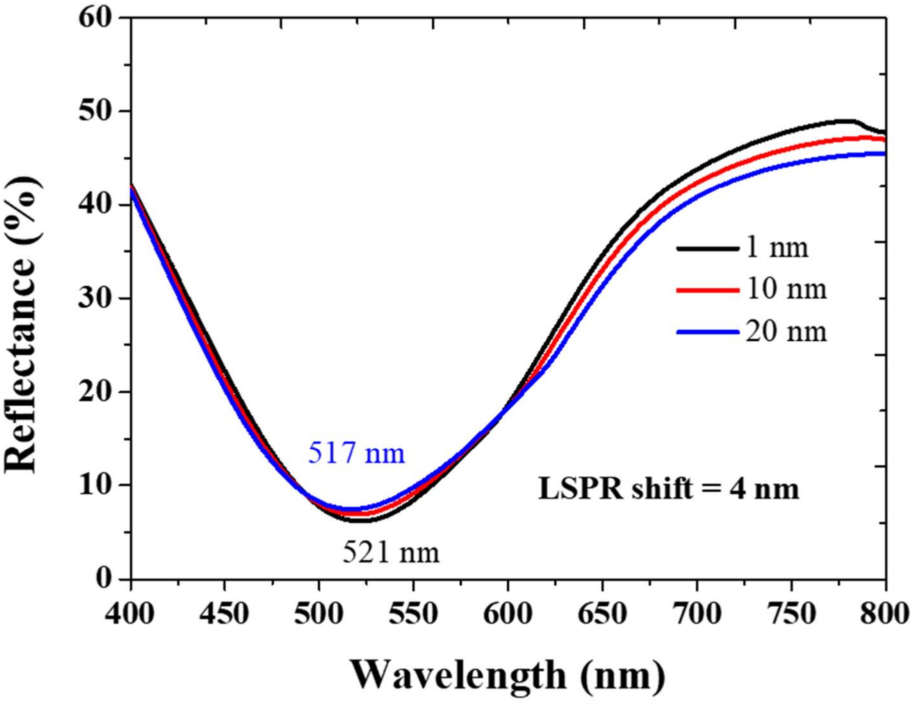

Figure S5. FDTD simulated reflectance spectra of dimple-textured Ga NPs with various interparticle gap sizes.
